# Long-Term Prognosis after ST-Elevation Myocardial Infarction in Patients with Premature Coronary Artery Disease

**DOI:** 10.3390/jpm14030231

**Published:** 2024-02-22

**Authors:** Lidija Savic, Igor Mrdovic, Milika Asanin, Sanja Stankovic, Ratko Lasica, Gordana Krljanac, Damjan Simic, Dragan Matic

**Affiliations:** 1 Faculty of Medicine, University of Belgrade, 11000 Beograd, Serbia; igormrd@gmail.com (I.M.); masanin2013@gmail.com (M.A.); drlasica@gmail.com (R.L.); gkrljanac@gmail.com (G.K.); dragan4m@gmail.com (D.M.); 2 Cardiology Intensive Care Unit & Cardiology Clinic, Emergency Hospital, University Clinical Center of Serbia, 11000 Belgrade, Serbia; simicdamjan@hotmail.com; 3 Center for Medical Biochemistry, Emergency Hospital, University Clinical Center of Serbia, 11000 Belgrade, Serbia; sstankovic2013@gmail.com

**Keywords:** premature coronary artery disease, ST-elevation myocardial infarction, prognosis, predictors

## Abstract

Background: A significant percentage of younger patients with myocardial infarction have premature coronary artery disease (CAD). The aims of this study were to analyze all-cause mortality and major adverse cardiovascular events (MACEs cardiovascular death, non-fatal reinfarction, stroke, target vessel revascularization) during eight-year follow-up in patients with ST-elevation myocardial infarction (STEMI) and premature CAD. Method: We analyzed 2560 STEMI patients without previous CAD and without cardiogenic shock at admission who were treated with primary PCI. CAD was classified as premature in men aged <50 years and women <55 years. Results: Premature CAD was found in 630 (24.6%) patients. Patients with premature CAD have fewer comorbidities and better initial angiographic findings compared to patients without premature CAD. The incidence of non-fatal adverse ischemic events was similar to the incidence in older patients. Premature CAD was an independent predictor for lower mortality (HR 0.50 95%CI 0.28–0.91) and MACEs (HR 0.27 95%CI 0.15–0.47). In patients with premature CAD, EF < 40% was the only independent predictor of mortality (HR 5.59 95%CI 2.18–8.52) and MACEs (HR 4.18, 95%CI 1.98–8.13). Conclusions: Premature CAD was an independent predictor for lower mortality and MACEs. In patients with premature CAD, EF < 40% was an independent predictor of eight-year mortality and MACEs.

## 1. Introduction

Despite major advances in the prevention and treatment of atherosclerosis, the incidence of coronary artery disease (CAD) is increasing in many regions of the world and is starting at an earlier age [1,2,3,4]. A significant percentage of younger patients with acute myocardial infarction (AMI) have premature CAD [5,6]. There is no universal definition of premature CAD [3,6,7,8,9,10]. Some authors have used an age cut-off of 45 years in men and 55 years in women [11], whereas other authors have used an age cut-off of 65 years [10]. Recently, an age cut-off of 50 years for men and 55 years for women has been suggested for defining premature CAD [7,10,12,13,14].

The literature data indicate that premature CAD is present in around 30% of patients diagnosed with CAD [11]. Patients with premature CAD are a population of particular concern because they may have a poor long-term outcome with a high likelihood of recurrence of the ischemic event [1,8,15]. These patients generally have different risk factors compared to older patients (especially more frequent smoking, family history, and dyslipidemia), but also other “unconventional” risk factors for coronary disease, such as psychosocial stress, obesity, use of psychoactive substances, etc. Also, more often than older patients, these patients have congenital or acquired disorders of hemostasis that can lead to arterial thrombosis and, consequently, to acute coronary syndrome (ACS) [3,5,11,15,16,17,18].

Compared to elderly patients, younger patients (therefore, also patients with premature CAD) have a better prognosis after MI in terms of lower mortality, but it has often been reported that the occurrence of recurrent non-fatal ischemic events does not differ significantly compared to the population of older patients [1,6,7,8]. ST-segment elevation myocardial infarction (STEMI) is more often the first manifestation of coronary disease in patients with premature CAD than is the case with the older population, where the first presentation of coronary disease is more often NSTEMI (non-STEMI) or unstable angina pectoris [9]. Also, young patients with STEMI have a poorer prognosis compared to young patients with NSTEMI, especially in short-term follow-up [19,20]. Primary percutaneous coronary intervention (pPCI) with stent implantation is currently considered the preferred treatment option for patients with ST-segment elevation myocardial infarction (STEMI) [21]. Many of the earlier analyses dealing with the prognosis in patients with premature CAD and AMI also included patients with STEMI and NSTEMI, as patients were not all treated with invasive procedures [7]. To the best of our knowledge, so far, there are not many studies in the literature that have analyzed the long-term prognosis of patients with STEMI who were treated with pPCI and who had premature CAD.

The aims of this study were as follows: (1) to analyze and compare all-cause mortality and the incidence of major adverse cardiovascular events (MACEs) during eight-year follow-up in patients with STEMI and premature and non-premature CAD; (2) to define independent predictors for eight-year all-cause mortality and MACEs in patients with premature CAD.

## 2. Materials and Methods

### 2.1. Study Population, Data Collection, and Definitions

In the present study, we included 2560 consecutive patients without previous coronary artery disease (CAD), hospitalized between December 2005 and January 2012, who were included in the prospective University Clinical Center of Serbia STEMI Register. The purpose of the prospective University Clinical Center of Serbia STEMI Register has been published previously [21].

**Figure 1:**
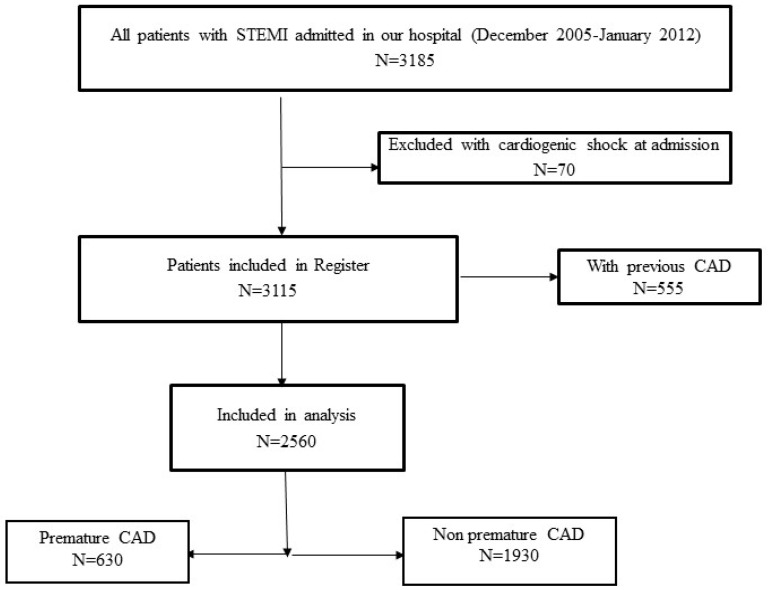
The flowchart of the patient selection.

The objective of the register is to gather data on the management and short- and long-term outcomes of patients with STEMI treated with primary PCI in the center. All consecutive STEMI patients aged 18 or older who were admitted to the Coronary Care Unit after being treated with pPCI in the center were included in the register. All included patients received written information about their participation in the register and the long-term follow-up, and their verbal and written consent was obtained. Patients with cardiogenic shock at admission were excluded from the register, and for the purpose of this study, we excluded patients with previous CAD. The flowchart of patient selection is presented in Figure 1.

Coronary angiography was performed via the femoral approach. Primary PCI and stenting of the infarct-related artery (IRA) were performed using the standard technique. Loading doses of aspirin (300 mg) and clopidogrel (600 mg) were administered to all patients before pPCI. Selected patients were also given the GP IIb/IIIa receptor inhibitor during the intervention. Flow grades were assessed according to the thrombolysis in myocardial infarction (TIMI) criteria. After pPCI, patients were treated according to the current guidelines.

Demographic, baseline clinical, laboratory, angiographic, and procedural data were collected and analyzed. Premature CAD was defined as the presence of CAD in men <50 years and in women <55 years. An echocardiographic examination was performed in the first three days after intervention (pPCI). The left ventricular ejection fraction (EF) was assessed according to the biplane method. We classified EF as preserved (EF ≥ 50%), moderately reduced (EF 40–49%), and reduced (EF < 40%). Baseline kidney function at admission was assessed using the Modification of Diet in Renal Disease equation, and a value below 60 mL/min/m^2^ was considered to be reduced kidney function. Obesity was defined as body mass index ≥ 30 kg/m^2^. Anemia was defined (according to the WHO criteria) as a baseline hemoglobin level below 120 g/L in men and below 110 g/L in women.

Patients were followed up at eight years after enrolment in the register. Follow-up data were obtained through telephone interviews and outpatient visits. We analyzed all-cause mortality and the composite endpoint: major adverse cardiovascular events (MACEs), which included cardiovascular death, non-fatal reinfarction, non-fatal ischemic stroke, and target vessel revascularization (TVR). The patients’ cause of death was obtained from death certificates or discharge forms (if the patient was hospitalized). Cardiovascular death included any death due to proximate cardiac cause (myocardial infarction, low-output heart failure, fatal arrhythmia, sudden death) and death caused by non-coronary vascular causes, such as cerebrovascular disease [21]. Non-fatal recurrent myocardial infarction was defined according to the Fourth Universal Definition for Myocardial Infarction [22]. Target vessel revascularization was defined as ischemia-driven percutaneous revascularization of the target vessel performed for restenosis or other complications. Stroke was defined as a new onset of focal or global neurological deficit lasting more than 24 h. Computed tomography was used to diagnose (ischemic) stroke. The emergency hospital neurologist was responsible for the diagnosis and treatment of stroke [21].

### 2.2. Ethics

The study protocol was approved by the ethics committee of the University of Belgrade, Faculty of Medicine (approval number 470/II-4, 21 February 2008). The study was conducted in accordance with the principles set forth in the Helsinki Declaration. Written informed consent was obtained from all patients for their participation in the register.

### 2.3. Statistical Analysis

Categorical variables were expressed as frequency and percentage, while continuous variables were expressed as the median (med), with 25th and 75th quartiles (IQR). Analysis for normality of data was performed using the Kolmogorov–Smirnov test. Baseline differences between groups were analyzed using the Mann–Whitney test for continuous variables and the Pearson χ^2^ test for categorical variables. The Kaplan–Meier method was used to construct the probability curves for eight-year mortality and the incidence of MACEs, while the difference between patients with and without premature CAD was tested with the Log-Rank test. The Cox proportional hazard model (backward method, with *p* < 0.10 for entrance into the model) was used to identify univariable and multivariable predictors for the occurrence of eight-year all-cause mortality and MACEs. Two-tailed *p*-values of less than <0.05 were considered statistically significant. We used the SPSS statistical software, version 19, for statistical analysis (SPSS Inc., Chicago, IL, USA).

## 3. Results

Of the 2560 patients analyzed, 630 (24.6%) patients had premature CAD. The mean age of all analyzed patients was 59 (51, 68) years, the mean age of patients with premature CAD was 45 (41, 48) years, and the mean age of patients with non-premature CAD was 62 (57, 71) years. As compared with patients with non-premature CAD, patients with premature CAD had fewer comorbidities; they were more likely to be smokers, to have hyperlipidemia, and to have a family history of CAD; they presented less often with heart failure, atrial fibrillation, and complete atrioventricular (AV) block; they had a shorter period of time from symptom onset to presentation at the hospital; they less often had multivessel disease on the initial coronary angiogram. Procedural characteristics did not differ among the analyzed groups. Pre-discharge EF was significantly higher in patients with premature CAD. Baseline characteristics, laboratory, angiographic, and procedural characteristics, in-hospital mortality, and therapy at discharge in patients with and without premature CAD are presented in Table 1.

At eight-year follow-up, all-cause mortality and MACEs were registered in a total of 172 (7.3%) patients and 391 (15.2%) patients, respectively. Causes of mortality were predominantly cardiovascular, while non-cardiovascular causes of death (cancer, ileus, and pneumonia) were registered in a total of 13 patients (5.1% of all deaths).

Patients with premature CAD had lower all-cause mortality and lower composite endpoint MACEs. Non-fatal recurrent infarction was related to a new lesion site in 39 (66.6%) patients with premature CAD and in 79 (46.7%) patients with non-premature CAD, *p* = 0.001.

All-cause mortality and MACEs in patients with premature CAD and in patients with non-premature CAD are presented in Table 2.

**Figure 2:**
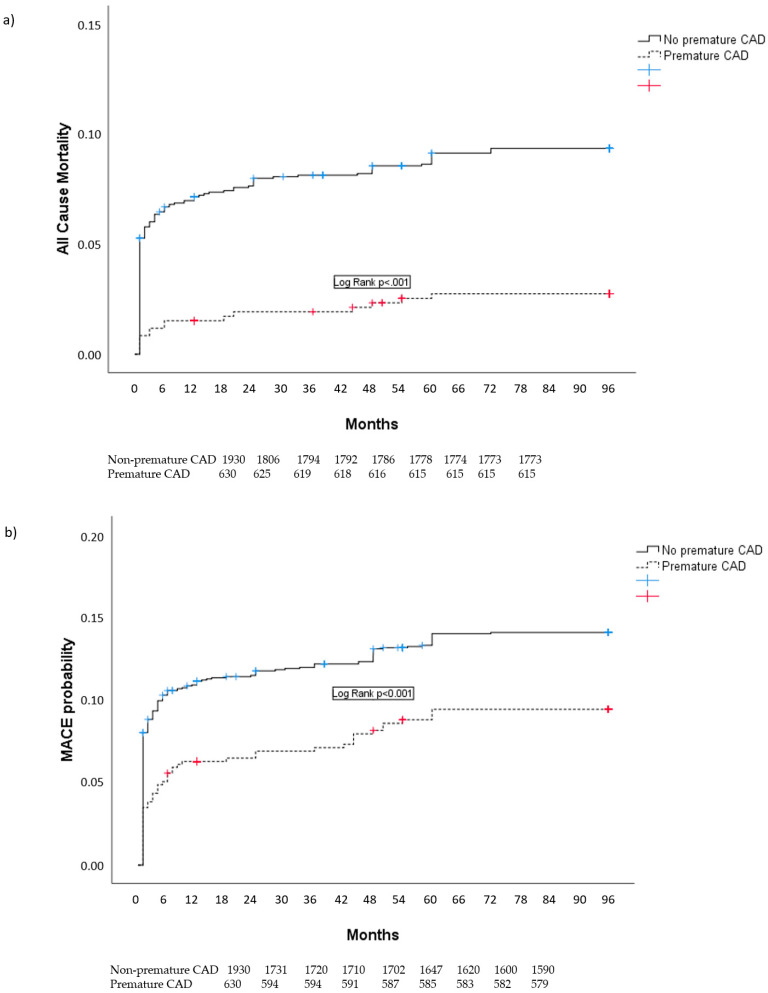
Kaplan–Meier curves showing all-cause mortality (curve (**a**)) and MACEs (curve (**b**)) in patients with premature and non-premature CAD. CAD=coronary artery disease; MACE=major adverse cardiovascula event (included cardiovascular death, non fatal recurrent infarction, non-fatal stroke and target-vessel revascularization).

Kaplan–Meier curves showing eight-year all-cause mortality and MACEs are presented in Figure 2.

After adjustment for confounders, premature CAD was associated with lower rates of all-cause mortality and MACEs in eight-year follow-up. This is presented in Table 3.

Independent predictors for eight-year all-cause mortality and MACEs in patients with premature CAD are presented in Table 4.

In patients with non-premature CAD, independent predictors for eight-year all-cause mortality were as follows: EF < 40% (HR 6.07, 95%CI 3.69–9.,97, *p* < 0.001), EF 40–49% (HR 2.31, 95%CI (1.14–3.37, *p* = 0.001), postprocedural flow TIMI < 3 (HR 2.16, 95%CI 1.39–3.45, *p* = 0.005), CKD (HR 1.72, 95%CI 1.19–2.49, *p* = 0.005), and Killip class >1 at admission (HR 1.68, 95%CI 1.15–2.45, *p* < 0.001), and independent predictors for eight-year MACEs were as follows: (older) age (HR 1.02–95%CI 1.01–1.04, *p* < 0.001), EF <40% (3.44, 95%CI 2.38–3.98, *p* < 0.001), EF 40–49% (HR 1.71, 95%CI 1.21–2.40, *p* = 0.002), postprocedural flow TIMI < 3 (HR 2.30, 95%CI 1.50–3.04), *p* < 0.001), and Killip class >1 at admission (HR 1.50, 95%CI 1.09–2.06, *p* = 0.034).

## 4. Discussion

Our results of the analysis of the STEMI Register showed about one-fourth of patients with premature CAD. Patients with premature CAD have significantly fewer comorbidities and better initial angiographic findings compared to patients without premature CAD. During eight-year follow-up, mortality and composite endpoint MACEs were significantly lower in patients with premature CAD compared to patients with non-premature CAD. The incidence of non-fatal recurrent ischemic events was not significantly different between these two groups of patients. In patients with premature CAD, only EF < 40% was an independent predictor of all-cause mortality and MACEs during eight-year follow-up.

### 4.1. The Incidence and Risk Factors of Premature CAD

The percentage of patients with premature CAD in our study is in keeping with the data from the literature, which states that the percentage of patients with premature CAD ranges between 20% and 30%, depending on the analyzed patient population as well as the age cut-off used for defining premature CAD [7,9,10,11,16]. Since it is known that CAD generally occurs later in women, we used a different age cut-off for defining premature CAD in men and women in our analysis [7,12,13]. In our population of patients with premature CAD, 25% of the patients were women, which is in keeping with data from the literature, where the reported percentage of women with premature CAD is around 20%–30% [7,23]. A higher prevalence of smoking, hyperlipidemia, and existing family history of coronary disease in patients with premature CAD, and fewer comorbidities (primarily diabetes mellitus, hypertension, CKD) is also a common finding in all analyses dealing with these patients [2,6,7].

### 4.2. Prognosis in Patients with Premature CAD

The relatively small number of studies analyzing patients with STEMI treated with primary PCI who have premature CAD makes it difficult to directly compare our results with those of other authors. It is well known that older age is one of the most important predictors of ACS outcomes, i.e., that younger age is a predictor of a more favorable prognosis [6,7,19]. However, we should not forget that myocardial infarction in (young) patients with premature CAD is an aggressive disease with a relatively high rate of recurrence [10].

In a study by Pinxterhuis et al., patients with premature CAD and the first presentation of coronary disease—STEMI and/or NSTEMI—were analyzed. In this study, it was found that premature CAD is a predictor of favorable prognosis during two-year follow-up, but also that the occurrence of non-fatal recurrent ischemic events does not differ between the group with premature CAD and the group without premature CAD [7], which is identical to our findings.

The results of a study by Lv et al., which presents an analysis of data from the register of patients with STEMI and NSTEMI, are similar. Significantly lower mortality and occurrence of MACEs were found during two-year follow-up in patients aged ≤45 years, as compared with patients aged >45 years. The incidence of non-fatal recurrent infarction did not differ between the two analyzed groups. Also, there was a “tendency” toward a lower incidence of non-fatal ischemic stroke in patients aged ≤45 years [6].

In an analysis of the Norwegian Myocardial Infarction Registry, it was found that the rate of AMI was low among people < 45 years old; however, almost 1 in 10 of these patients experienced a new cardiovascular event, while 4% of patients died during the 2.4 years of follow-up [23]. In our study, the percentage of mortality and occurrence of MACEs is lower compared to the analysis of this registry, and the differences are probably due to the different patient populations that were analyzed (STEMI/NSTEMI), as well as the differently defined age cut-off.

In a prospective register analysis by Wittlinger et al., patients with STEMI and NSTEMI were included. In this study, younger patients under the age of 40 years had better two-year survival compared to those older than 40 years, and the highest number of deaths occurred in the first 10 days after the initial event. In our study as well, the highest percentage of adverse events occurred in the first days/months of follow-up. However, unlike our study, this study also included patients with STEMI who were treated with both thrombolysis and rescue PCI [3].

In a study by Noaman et al., data from the register of patients who underwent PCI due to chronic and acute coronary syndrome were analyzed. Patients younger than 45 years had a lower 12-year mortality compared to middle-aged (46–65 years) and elderly patients (>65 years). The age of <45 years was an independent predictor of better long-term survival compared to middle age (46–65 years) [19].

The results of a study by Yang et al. analyzing young patients with MI showed no difference in one-year outcome and long-term mortality (more than 11 years) between patients younger than 40 years and patients aged 40–51 years [16]. However, the fact remains that this study was generally focused on young patients, i.e., patients with premature CAD. In our study, in the subgroup of patients with premature CAD, age was not a predictor of eight-year mortality and MACEs.

In a study analyzing patients with premature CAD who underwent their first PCI due to STEMI or NSTEMI, it was found that patients with premature CAD had lower mortality but a higher incidence of stent thrombosis and repeat revascularization than patients with non-premature CAD during three-year follow-up [13]. In our study, the incidence of non-fatal adverse ischemic events during follow-up did not differ between the analyzed groups of patients, while the cause of non-fatal recurrent infarction in our patients was predominantly the appearance of new lesions in the coronary arteries, which was more frequent in comparison with patients with non-premature CAD.

According to the available literature data, up to 30% of younger patients develop new ischemic events after ACS in the first five years of follow-up, i.e., before the age of 60, and angiographic findings show that most often, new lesions are found in the coronary arteries, which is different from the older population [1,8].

### 4.3. Independent Predictors of Mortality and MACEs in Patients with Premature CAD

Predictors of mortality and of the occurrence of MACEs in younger patients with ACS (which also include patients with premature CAD) differ depending on the study method, i.e., patient selection, as well as the type of ACS: STEMI, NSTEMI, or unstable angina [6,19,24]. Our finding that reduced EF is an independent predictor of mortality and MACEs in patients with premature CAD is in concordance with the findings of studies that analyzed the prognosis in younger patients (which also includes patients with premature CAD).

In the study by Lv et al., EF, creatinine level, and education level were independent predictors of two-year mortality in AMI patients aged ≤45 [6]. In the study by Noaman et al., it was found that independent predictors of 12-year mortality in patients aged ≤45 years with acute coronary syndrome are the following: poor angiographic findings, LM coronary intervention, severe CKD, EF < 30%, chronic lung disease, and diabetes mellitus [19]. Unlike our study, in the cited study, only 40% of patients had STEMI [19], and patients with earlier CAD were also included, which can explain the differences in the results. In a study by Liang et al. in which patients with STEMI were analyzed, it was found that only diabetes mellitus was an independent predictor of the 30-month occurrence of MACEs in patients younger than 45 years [18]. In this study, only demographic characteristics, risk factors, and angiographic findings were analyzed, and echocardiographic parameters were not presented/analyzed [18].

In comparison with patients with premature CAD, in patients with no-premature CAD, there were more independent predictors for both eight-year all-cause mortality and MACEs. In older patients, independent predictors for all-cause mortality and MACEs were EF but also clinical and procedural characteristics. All these predictors are well-known predictors of adverse events in patients with STEMI [25]. Different predictors for all-cause mortality and MACEs in patients with premature CAD and older patients can be explained by different baseline comorbidities but also by different procedural characteristics that exist between these two groups.

### 4.4. Clinical Implications

The results of our study can supplement existing knowledge regarding the prognosis of patients with STEMI caused by premature CAD who were treated with primary PCI. The prognosis of patients with premature CAD, although more favorable than the prognosis of older patients, is still not completely favorable, and the occurrence of recurrent non-fatal myocardial infarction, caused predominantly by new lesions in the coronary arteries, indicates that more aggressive secondary prevention of coronary disease is necessary, as well as more frequent follow-up during monitoring, so as to improve the prognosis and thus the quality of life [1,5,10,11,19,24]. In certain patients, investigation is indicated in terms of the existence of congenital and acquired thrombophilic conditions [7]. EF is the measure most commonly used to assess left ventricular systolic function. It is well known that EF is one of the most important predictors of prognosis after STEMI, and patients with reduced EF have a significantly worse prognosis compared to patients with preserved EF [25]. The main reason for ventricular remodeling and reduced left ventricular systolic function (i.e., reduced EF) in patients with STEMI is the large zone of myocardial ischemia that leads to the necrosis of cardiomyocytes. Establishing normal blood flow through the infarcted artery (with primary PCI or, less often, with thrombolytic therapy) leads to a reduction in the myocardial necrotic zone, improves LV function, and decreases LV remodeling [26,27]. Myocardial stunning is another mechanism that can cause left ventricular systolic dysfunction in patients treated with successful and timely reperfusion therapy (TIMI flow 3 through infarct-related artery). Myocardial necrosis and stunning lead to subsequent myocardial inflammation, hypertrophy, and fibrosis. In addition, different neurohumoral (such as renin–angiotensin–aldosterone system, sympathetic system, etc.) and mechanical changes contribute to decreasing in the systolic function of the left ventricle [26,28]. Before discharge from the hospital, and especially during follow-up, special attention should be paid to young patients with premature CAD and reduced EF. In these patients, apart from secondary prevention of coronary disease, it is extremely important to include and titrate up to the target doses of therapy as early as possible, as this has been proven to have a favorable effect on the prognosis in the presence of reduced EF after myocardial infarction [28].

### 4.5. Study Limitations

A number of limitations to our study should be mentioned. The study is unicentric and observational, but it is controlled, prospective, and has included consecutive patients. This can limit possible selection bias. Patients included in the study were hospitalized between 2005 and 2012. Patients with cardiogenic shock at admission were excluded from our register. All patients were treated with clopidogrel. There were no patients treated with more recently developed antiplatelet drugs (ticagrelor and prasugrel were not available for routine administration to patients at the time of their entry into the register). This may have influenced the patients’ prognosis, i.e., reduced the occurrence of cardiovascular mortality or the incidence of non-fatal ischemic events. Coronary angiography and concomitant PCI were performed via the femoral approach. The radial approach was not used in routine clinical practice at the time of the patients’ enrolment into the register. We did not analyze “unconventional” risk factors for coronary disease in patients with premature CAD (congenital or acquired thrombophilia), such as the presence of menopause in women, the possible use of hormone replacement therapy, socioeconomic status, as well as possible substance abuse in patients with premature CAD [10]. There are no data on follow-up echocardiographic examinations to show whether there has been a certain degree of recovery or deterioration in the myocardial contractility. The study was not designed to evaluate whether changing pharmacological treatment during follow-up would have an impact on long-term outcomes in the analyzed patients.

## 5. Conclusions

About 25% of the analyzed patients with STEMI had premature CAD. As compared with older patients, those with premature CAD had lower eight-year mortality and MACEs. The incidence of non-fatal adverse ischemic events was similar to the incidence in older patients. Recurrent non-fatal myocardial infarction in patients with premature CAD was predominantly caused by the appearance of new lesions in the coronary arteries. In patients with premature CAD, only EF < 40% was an independent predictor of eight-year all-cause mortality and MACEs. These findings indicate the great importance of the need for secondary prevention of coronary disease in patients with premature CAD, as well as early inclusion and titration to target doses of therapy, which has been proven to have a beneficial effect on prognosis in the presence of reduced EF after myocardial infarction.

## Figures and Tables

**Table 1:** Baseline clinical, laboratory, angiographic, procedural characteristics, therapy at discharge, and in-hospital mortality of the study patients.

Characteristics	Premature CAD	Non-Premature CAD	*p*-Value
	N = 630	N = 1930	
Age, years med (IQR)	45 (41, 48)	62 (56, 71)	<0.001
Female, *n* (%)	162 (25.7)	522 (27)	0.177
Hypertension, *n* (%)	293 (46.5)	1346 (69.7)	<0.001
HLP, *n* (%)	413 (65.5)	1116 (57.8)	<0.001
Smoking, *n* (%)	485 (76.9)	922 (47.8)	<0.001
Family history, *n* (%)	314 (49.8)	542 (28)	<0.001
Pain duration, hours med (IQR)	2 (1, 4)	3 (1.5, 4)	<0.001
Atrial fibrillation on initial ECG, *n* (%)	13 (2.1)	145 (7.5)	<0.001
Complete AV block, *n* (%)	13 (2.1)	101 (5.2)	0.005
Killip class > 1, *n* (%)	48 (7.6)	254 (13.2)	0.001
Systolic BP at admission, med (IQR)	130 (120, 150)	140 (120, 150)	<0.001
Heart rate at admission med (IQR)	80 (70, 90)	76 (68, 89)	0.059
BBB on initial ECG, *n* (%)	16 (2.6)	71 (3.6)	0.559
Multivessel disease, *n* (%)	229 (36.3)	1130 (58.5)	<0.001
3-vessel disease, *n* (%)	75 (11.9)	520 (26.9)	<0.001
LM stenosis, *n* (%)	13 (2.1)	137 (7.1)	<0.001
LAD culprit vessel, *n* (%)	249 (39.5)	765 (39.7)	0.583
Preprocedural flow TIMI 0, *n* (%)	440 (69.8)	1328 (68.8)	0.379
Postprocedural flow TIMI < 3, *n* (%)	16 (2.5)	81 (4.2)	0.067
Stent implanted, *n* (%)	610 (96.7)	1835 (95.1)	0.067
Total stent length, med (IQR)	23 (18, 25)	24 (19, 26)	0.204
Acute stent thrombosis, *n* (%)	10 (1.5)	18 (0.9)	0.162
CK-MB med (IQR)	2254 (1051, 3898)	1925 (1067, 3391)	<0.001
Troponin I, med (IQR)	93.2 (34.1, 183.1)	90.7 (33.7, 172)	0.001
EF, med (IQR)	50 (42, 58)	47 (40, 55)	<0.001
EF < 40%, *n* (%)	63 (10%)	620 (24.2)	<0.001
EF 40–49%, *n* (%)	155 (24.6)	953 (37.2)	<0.001
EF ≥ 50%, *n* (%)	402 (63.8)	986 (38.5)	<0.001
Diabetes, *n* (%)	57 (9.1)	400 (20.7)	<0.001
CKD, *n* (%)	8 (1.2)	358 (18.5)	<0.001
Obesity, *n* (%)	124 (19.7)	287 (14.9)	0.018
Anemia, *n* (%)	18 (2.8)	164 (8.5)	<0.001
Previous stroke, *n* (%)	8 (1.3)	81 (4.2)	<0.001
Therapy at discharge *			
Beta blockers, *n* (%)	611 (93)	1848 (95.7)	0.150
ACE inhibitors, *n* (%)	539 (85.6)	1739 (90.1)	0.003
Statin, *n* (%)	607 (96.3)	1902 (98.5)	0.139
Diuretic, *n* (%)	62 (9.9)	306 (15.8)	<0.001
Calcium antagonist, *n* (%)	11 (1.7)	70 (3.6)	0.009
Amiodarone, *n* (%)	13 (2)	52 (3.1)	0.133
In-hospital mortality, *n* (%)	4 (0.6)	80 (4.1)	<0.001

* All patients were on aspirin and clopidogrel CAD = coronary artery disease; med = median; IQR = interquartile range; EF = left ventricular ejection fraction; HLP = hyperlipidemia; AV = atrioventricular; HF = heart failure; BP = arterial blood pressure; BBB = bundle branch block on admission ECG; CKD = chronic kidney disease; PAD = peripheral artery disease; LM = left main artery; LAD = left anterior descendent; CK-MB = creatine kinase MB isoform.

**Table 2:** Mortality and composite endpoint MACEs in study patients.

Event	Premature CAD	Non-Premature CAD	*p*-Value
	N = 630	N = 1930	
30 days			
All-cause mortality, *n* (%)	5 (0.7)	93 (4.8)	<0.001
MACE, *n* (%)	30 (4.7)	157 (8.1)	<0.001
Cardiovascular death, *n* (%)	5 (0.7)	93 (4.8)	<0.001
Non-fatal recurrent infarction, *n* (%)	19 (3)	64 (3.3)	0.540
TVR, *n* (%)	19 (3)	64 (3.3)	0.540
Non-fatal stroke, *n* (%)	0	15 (0.7)	0.085
1 year			
All-cause mortality, *n* (%)	9 (1.4)	126 (6.5)	<0.001
MACE, *n* (%)	36 (5.6)	205 (10.6)	0.022
Cardiovascular death, *n* (%)	9 (1.4)	105 (5.4)	<0.001
Non-fatal recurrent infarction, *n* (%)	27 (4.3)	98 (5.1)	0.362
TVR, *n* (%)	34 (5.4)	106 (5.5)	0.360
Non-fatal stroke, *n* (%)	2 (0.3)	28 (1.4)	0.080
MACE, *n* (%)	36 (5.6)	205 (10.6)	0.022
8 years			
All-cause death, *n* (%)	15 (2.4)	157 (8.1)	<0.001
MACE, *n* (%)	51 (8)	340 (17.6)	<0.001
Cardiovascular death, *n* (%)	13 (2)	146 (7.6)	<0.001
Non-fatal recurrent infarction, *n* (%)	51 (8.1)	168 (8.7)	0.726
TVR, *n* (%)	58 (9.2)	169 (8.6)	0.585
Non-fatal stroke, *n* (%)	7 (1.1)	51 (2.6)	0.089

CAD = coronary artery disease; MACEs = major adverse cardiovascular events; TVR = target vessel revascularization.

**Table 3:** Univariable and multivariable analysis for all-cause mortality and MACEs in all analyzed patients (Cox regression model).

	All-Cause Mortality		MACE	
	HR (95%CI)	*p*-Value	HR (95%CI)	*p*-Value
Univariable analysis				
Premature CAD	0.50 (0.28–0.91)	<0.001	0.27 (0.15–0.47)	<0.001
Killip > 1 at admission	5.04 (3.78–6.89)	<0.001	7.02 (5.21–9.45)	<0.001
Atrial fibrillation at admission	4.58 (3.12–6.59)	<0.001	4.69 (3.22–6.83)	<0.001
CKD	4.90 (3.59–6.69)	<0.001	5.36 (3.88–7.41)	<0.001
Postprocedural TIMI < 3	3.36 (2.43–5.42)	<0.001	7.52 (5.14–11.01)	<0.001
3-vessel disease	1.56 (1.14–2.14)	0.005	2.05 (1.49–2.83)	<0.001
Ejection fraction %	0.89 (0.87–0.90)	<0.001	0.88 (0.87–0.89)	<0.001
Systolic blood pressure at admission, mmHg	0.99 (0.98–0.99)	0.024		
Complete AV block at admission			2.80 (1.93–4.07)	<0.001
Diabetes mellitus			2.0 (1.42–2.82)	<0.001
Multivariable analysis				
Premature CAD	0.48 (0.26–0.83)	0.001	0.50 (0.27–0.91)	0.026
CKD	2.36 (1.68–3.31)	<0.001	2.74 (1.94–3.89)	<0.001
Postprocedural TIMI < 3	2.14 (1.40–3.27)	<0.001	2.41 (1.56–3.72)	<0.001
Killip > 1 at admission	1.89 (1.26–2.58)	0.001	1.96 (1.35–2.85)	<0.001
Atrial fibrillation at admission	1.61 (1.08–2.41)	0.019	1.58 (1.04–2.38)	0.031
3-vessel disease			1.50 (1.04–1.90)	0.045
Ejection fraction %	0.91 (0.90–0.93)	<0.001	0.90 (0.88–0.92)	<0.001

EF = left ventricular ejection fraction; CKD = chronic kidney disease; CAD = coronary artery disease; TIMI = thrombolysis in myocardial infarction; AV = atrioventricular.

**Table 4:** Independent predictors for 8-year all-cause mortality and MACEs (Cox regression model) in patients with premature CAD.

	Univariate Analysis		Multivariable Analysis	
	HR (95%CI)	*p*-Value	HR (95%CI)	*p*-Value
All-cause mortality				
EF < 40%	8.28 (5.25–12.35)	<0.001	5.59 (2.18–8.52)	<0.001
EF 40–49%	2.04 (1.17–9.12)	0.025		
LM stenosis	7.26 (1.69–12.12)	0.009		
Baseline CKD	6.91 (3.25–16.8)	0.035		
Killip class > 1 at admission	6.29 (3.14–12.59)	<0.001		
Postprocedural TIMI < 3	6.09 (1.35–20.9)	0.019		
Atrial fibrillation at admission	3.58 (1.27–13.5)	0.001		
MACE				
EF < 40%	4.79 (2.39–8.74)	<0.001	4.18 (1.98–8.13)	<0.001
EF 40–49%	1.59 (1.11–2.91)	0.050		
Baseline CKD	3.79 (2.45–5.61)	0.001		
Postprocedural TIMI < 3	2.39 (1.17–7.49)	0.021		
Smoking	2.16 (1.19–5.08)	0.059		
Killp class > 1 at admission	1.99 (1.09–4.43)	0.049		
3-vessel disease	1.51 (1.01–2.67)	0.050		

MACEs = major adverse cardiovascular events; EF = left ventricular ejection fraction; CKD = chronic kidney disease; TIMI = thrombolysis in myocardial infarction; LM = left main coronary artery.

## Data Availability

The data presented in this study are available on request from the corresponding author.
